# Combined serum and synovial C-reactive protein tests: a valuable adjunct to the diagnosis of chronic prosthetic joint infection

**DOI:** 10.1186/s12891-021-04545-6

**Published:** 2021-08-09

**Authors:** Hai Wang, Leilei Qin, Jiawei Wang, Ning Hu, Wei Huang

**Affiliations:** 1 Department of Orthopaedics, The First Affiliated Hospital of Chongqing Medical University, Chongqing, 400016 China; 2 Department of Orthopaedics, Fuling Central Hospital of Chongqing City, Chongqing, 408099 China

**Keywords:** Chronic Periprosthetic joint infection, Diagnosis, Synovial fluid, C-reactive protein (CRP)

## Abstract

**Background:**

Diagnosis of periprosthetic joint infection (PJI), especially chronic PJI, is very confusing and challenging. The value of C-reactive protein (CRP) in infectious diseases has been recognized, but the diagnostic value of CRP in chronic PJI is unknown. Our aim was to investigate the diagnostic value of synovial CRP in chronic PJI and to explore the role of combined serum and synovial CRP in distinguishing chronic PJI from aseptic failure after knee and hip arthroplasties.

**Methods:**

We prospectively enrolled patients scheduled to have a revision surgery for chronic PJI or aseptic loosening from January 2019 to December 2020, in which synovial CRP was additionally measured along with routine preoperative diagnostic serum ((ESR, CRP) and synovial (PMN%) biomarkers. The receiver operating characteristic (ROC) curves and area under the curve (AUC) were analyzed for each biomarker to determine diagnostic efficacy.

**Results:**

There were no statistically significant differences between the infection (*n* = 39) and aseptic (*n* = 58) groups, including 61 hips and 36 knees. The synovial CRP levels were significantly higher in the infection group than in the aseptic group (median: 9.93 mg/l vs 3.58 mg/l; *p* < .001). The optimal cut-off value for detecting chronic PJI of Synovial fluid (SF) CRP was of 7.26 mg/l with a sensitivity of 84.62%, a specificity of 93.10%. The combined model I (Serum CRP > 10.2 mg/l OR SF CRP > 7.26 mg/l) had a negative predictive value (NPV) of 96.67%, and a sensitivity of 97.44%. The combined model II (Serum CRP > 10.2 mg/l AND Synovial CRP > 7.26 mg/l) led to a specificity of 1, and a positive predictive value (PPV) of 1.

**Conclusions:**

The present study demonstrated that the combination of serum and synovial CRP can be used as an adjunct to the diagnosis of chronic PJI.

**Supplementary Information:**

The online version contains supplementary material available at 10.1186/s12891-021-04545-6.

## Background

Prosthetic joint infection (PJI) is one of the most serious complications after total joint arthroplasties [1]. However, in some cases, it is difficult to distinguishing septic and aseptic failures after total joint arthroplasties, especially in patients with chronic PJI [2]. Identifying the diagnosis of chronic PJI is very challenging due to atypical symptoms, which may lead to infection with delayed healing, severe bone defects, joint dysfunction and even a higher risk of short-term mortality [3]. To date, there is still no “gold standard” tests or protocol for diagnosing chronic PJI [4]. Moreover, the distinction between septic and aseptic failures is critical because of the significant differences in treatment regimens and the serious adverse consequences of misdiagnosis for patients [5]. So, accurate and timely diagnosis of chronic PJI is a key step toward implementing an effective treatment.

Elevated serum inflammatory biomarkers, such as serum C-reactive protein (CRP) and erythrocyte sedimentation rate (ESR) may be the primary indications of chronic PJI due to atypical clinical symptoms such as joint effusion, pain, swelling, and redness. However, due to insufficient specificity, these tests are not sufficient to diagnose PJI alone [6]. More specific tests need to be supplemented, such as synovial fluid analysis, microbial culture, and histopathology. Among them, the detection of synovial fluid (such as IL-6, CD64, and alpha-defensin) has shown great attraction in recent studies [4, 7, 8]. CRP is one of the most widely used inflammatory markers in the identification of infectious diseases, which is synthesized primarily in liver hepatocytes but also by smooth muscle cells, macrophages, endothelial cells, lymphocytes, and adipocytes [9, 10]. Its physiological role is to bind to lysophosphatidylcholine expressed on the surface of dead or dying cells (and some types of bacteria) in order to activate the complement system via C1q [11]. C-reactive protein is a non-specific parameter for inflammation, and its levels increase during bacterial infection. And studies have shown that, CRP is deposited at sites of inflammation and tissue damage in both naturally occurring and experimental conditions [12]. Therefore, the deposition of CRP at the site of infection can be detected, especially in the liquid phase [13]. Recent studies have shown that measuring CRP levels in synovial fluid may be a valuable mean to improve the diagnosis of PJI [1, 14]. However, studies on synovial CRP were so far limited to small sample sizes, and studies on chronic PJI were insufficient.

In this study, we thus sought to (1) determine the utility of serum and synovial CRP in distinguishing between aseptic failure and chronic infections in patients undergoing revision surgery for failure of total joint arthroplasty, and (2) establish combined cut-off values of serum and synovial CRP in confirming chronic PJI.

## Methods

### Study design and inclusion and exclusion criteria

Patients scheduled to have a revision surgery for chronic infection of knee and hip arthroplasties or aseptic loosening of an implant were enrolled from January 2019 to December 2020. The approval of Institutional Review Board for collection of all patients’ samples was obtained. The patients were divided into “aseptic” and “infection” groups, according to the 2013 Musculoskeletal Infection Society (MSIS) criteria for the diagnosis of PJI [15]. The guidelines include major and minor diagnostic criteria, with the latter involving measurements of serum C-reactive protein (CRP) level and erythrocyte sedimentation rate (ESR), synovial fluid white blood-cell (WBC) count and neutrophil differential, culture, and leukocyte esterase testing (Supplementary Table 1).

The “aseptic group” was defined as patients who did not fulfill the definition of PJI and did not develop infection or undergo a reoperation for at least 1 year following the index arthroplasty. A postoperative infection was considered ‘chronic’ when PJI symptoms occurred beyond 6 weeks after implantation [16–18].

Patients with the following conditions were excluded: malignancy, rheumatism, renal failure, autoimmune disease, chronic infectious disease (such as human immunodeficiency virus or hepatitis C virus), patients with recent antibiotic use (less than 2 weeks), inadequate synovial fluid acquisition and patients developed infection within 1 year after aseptic revision.

### Samples and data collection

All blood samples were collected on the day of surgery. The synovial fluid of the knee was collected on the day the blood samples were collected and the synovial fluid of the hip was collected when the capsule was incised intraoperatively. All specimens were submitted for examination within 2 h after collection. To assess CRP levels, plasma and synovial fluid were stored in lithium-heparin vacuum collection tubes. The CRP was tested using a particle-enhanced turbidimetric immunoassay with a HITACHI 7600 Series Automatic Biochemical Analyzer (Hitachi, Tokyo, Japan) and diagnostic kit (DiaSys Diagnostic Systems GmbH, Shanghai, China). Synovial fluid was examined for WBCs and PMNs using a haematology analyzer (Symex XE-5000 haematology analyzer, Symex, Japan), and the synovial fluid was cultured for 14 days on Columbia agar, chocolate agar, and Schaedler agar. At least three suspected tissue specimens were obtained intraoperatively by a stationary surgeon for culture, and the tissue specimens were subjected to intraoperatively frozen section and histopathological examination.

### Statistical analysis

Statistical analysis was carried out using SPSS version 24. The data were presented as medians and interquartile ranges (IQRs). The independent sample t-test was used for the data conforming to the normal distribution, and the Mann-Whitney-U test was used for the data not conforming to the normal distribution. Receiver operating characteristic (ROC) curve analyses was used to assess the ability of serum and synovial fluid CRP concentration to determine the presence of PJI. Youden’s J statistic was used to determine optimum cut-off values for the diagnosis of chronic infection. The area under the curves (AUCs) of each test were compared using MedCalc 13.2.2 Software (MedCalc Software BV, Ostend, Belgium), and based on the cut-off values, the sensitivity, specificity, positive predictive value (PPV), and negative predictive value (NPV) of these makers were calculated from contingency Tables. A *p*-value ≤0.05 was considered statistically significant.

## Results

**Fig. 1:**
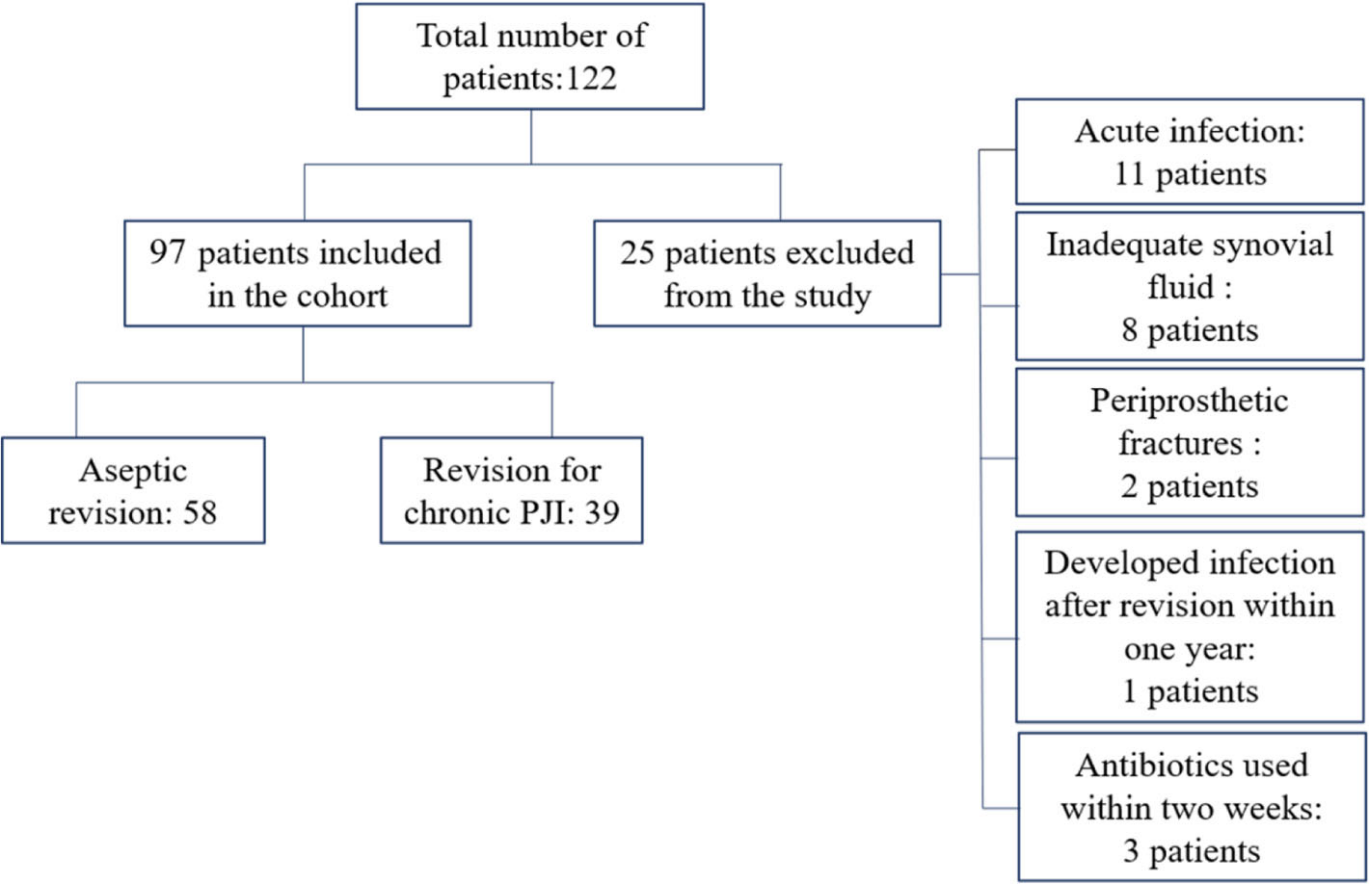
Patients Flow Chart

From January 2019 to December 2020, 122 patients with revision surgery after knee and hip arthroplasties in our department were screened, of which 97 were eligible. Reasons for exclusion include: acute infection within 6 weeks after primary arthroplasties (11, 44%), inadequate synovial fluid acquisition (8, 32%), revision surgery for periprosthetic fractures (2, 8%), local or intravenous antibiotics within 2 weeks (3, 12%) and 1 patient developed infection 273 days after aseptic revision. Fifty-eight patients were assigned to the “aseptic” group and 39 to the “infection” group (Fig. 1). The patients’ demographics were shown in Table 1. Data in Table 1 did not show a significant difference in Ages, Sex, Joint type and BMI between the groups. Mean time since prosthesis implantation was 9.87 years (SD 2.67) in the group with aseptic revision and 2.96 years (SD 1.05) in the group with infection (*p* < 0.01). In the infection group, the causative organism could be identified in 87.18% of the cases (34/39). The most commonly isolated pathogens were coagulase-negative staphylococci (*n* = 13, 38.24%), *S. aureus* (*n* = 11, 32.35%), *Staphylococcus epidermidis* (*n* = 3, 8.82%), Methicillin-resistant *S. aureus* (MRSA) (*n* = 2, 5.88%), *C. tropicalis* (n = 2, 5.88%), *Streptococcus agalactiae* (n = 2, 5.88%) and Carbapene-resistant *A. baumannii* (1, 2.95%).

**Table 1:** Demographic data for the study population. Variables are expressed as means (SDs) or absolute numbers and percentages

	aseptic (*n* = 58)	infected (*n* = 39)	*P* value
Age (years)			
MEAN	69.37	73.28	0.078^b^
SD	12.63	6.18	
Sex, n (%)			
Male	28 (48.28)	22 (56.41)	0.535^c^
Female	30 (51.72)	17 (43.59)	
Joint type, n (%)			
Hip	33 (56.90)	28 (71.79)	0.198^c^
Knee	25 (43.10)	11 (28.21)	
Height (cm)			
MEAN	161.28	161.95	0.680^b^
SD	7.78	7.98	
Weight (kg)			
MEAN	60.69	59.97	0.775^b^
SD	12.74	10.85	
BMI (kg/m2)			
MEAN	23.34	22.90	0.632^b^
SD	4.66	4.04	
Time Frame (year)			
MEAN	9.87	2.96	< 0.001^b/a^
SD	2.67	1.05	

“^a^” means statistically significant values “^b^” Independent-samples t-test. “^c^” Fisher’s exact test. Variables are expressed as mean ± SD (standard deviation), or numbers (percentage), BMI (Body Mass Index)

**Fig. 2:**
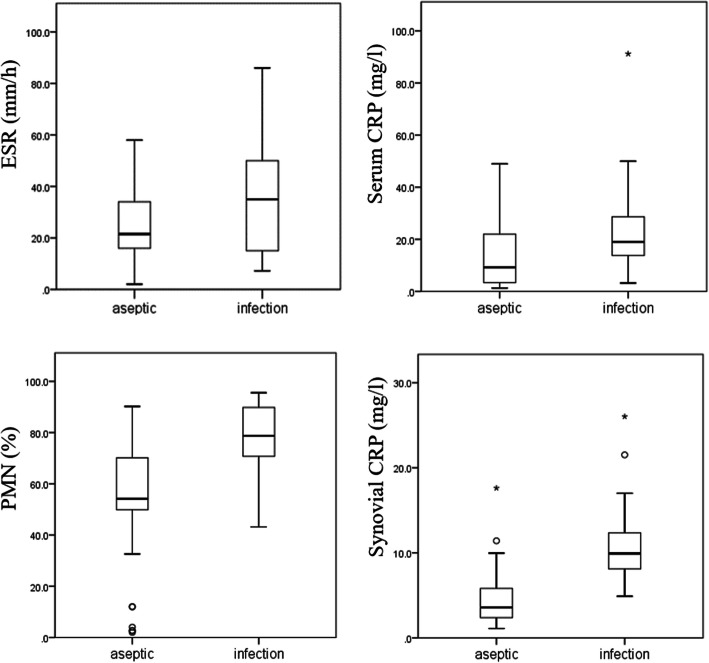
Boxplots of serum CRP, ESR, synovial CRP and PMN% levels in the two groups. The horizontal line represents the median level, the black box the interquartile range, the whiskers the minimum and maximum and the cross outliners

The median concentration of CRP in the synovial fluid for the infection group was 9.93 mg/l and was significantly higher (*p* < 0.001) than the aseptic group with a median concentration of 3.58 mg/l (Table 2). Serum CRP and synovial fluid percentage of polymorphonuclear neutrophils (PMN%) were significantly higher in the infection group than in the aseptic group (Table 2 and Fig. 2). While the ESR did not differ significantly between the two groups (*p* = .097). In addition, we performed a subgroup analysis of infected hip and knee arthroplasty, and there were no significant differences (Supplementary Table 2).

**Table 2:** Analysis of inflammatory markers in patients with infected and aseptic revision arthroplasty

Inflammatory marker	Hip + Knee		
	Aseptic (*n* = 58)	Infection (*n* = 39)	*P* value
ESR (mm/h)			
median	21.5	35	0.097^b^
P25, P75	(16.25, 34.0)	(15.0, 50.0)	
Serum CRP (mg/L)			
median	9.25	19	0.001^b, a^
P25, P75	(3.36, 21.15)	(13.8, 28.6)	
PMN% (%)			
median	54.16	78.75	< 0.001^#, a^
P25, P75	(49.85, 70.06)	(70.73, 89.81)	
Synovial CRP (mg/L)			
median	3.58	9.93	< 0.001^b,^ *
P25, P75	(2.40, 5.79)	(8.12, 12.35)	

*CRP* C-reactive protein, *ESR* erythrocyte sedimentation rate, *PMN%* Percentage of Polymorphonuclear Cells

“^a^” means statistically significant values; “^b^” Mann-Whitney-U test

**Fig. 3:**
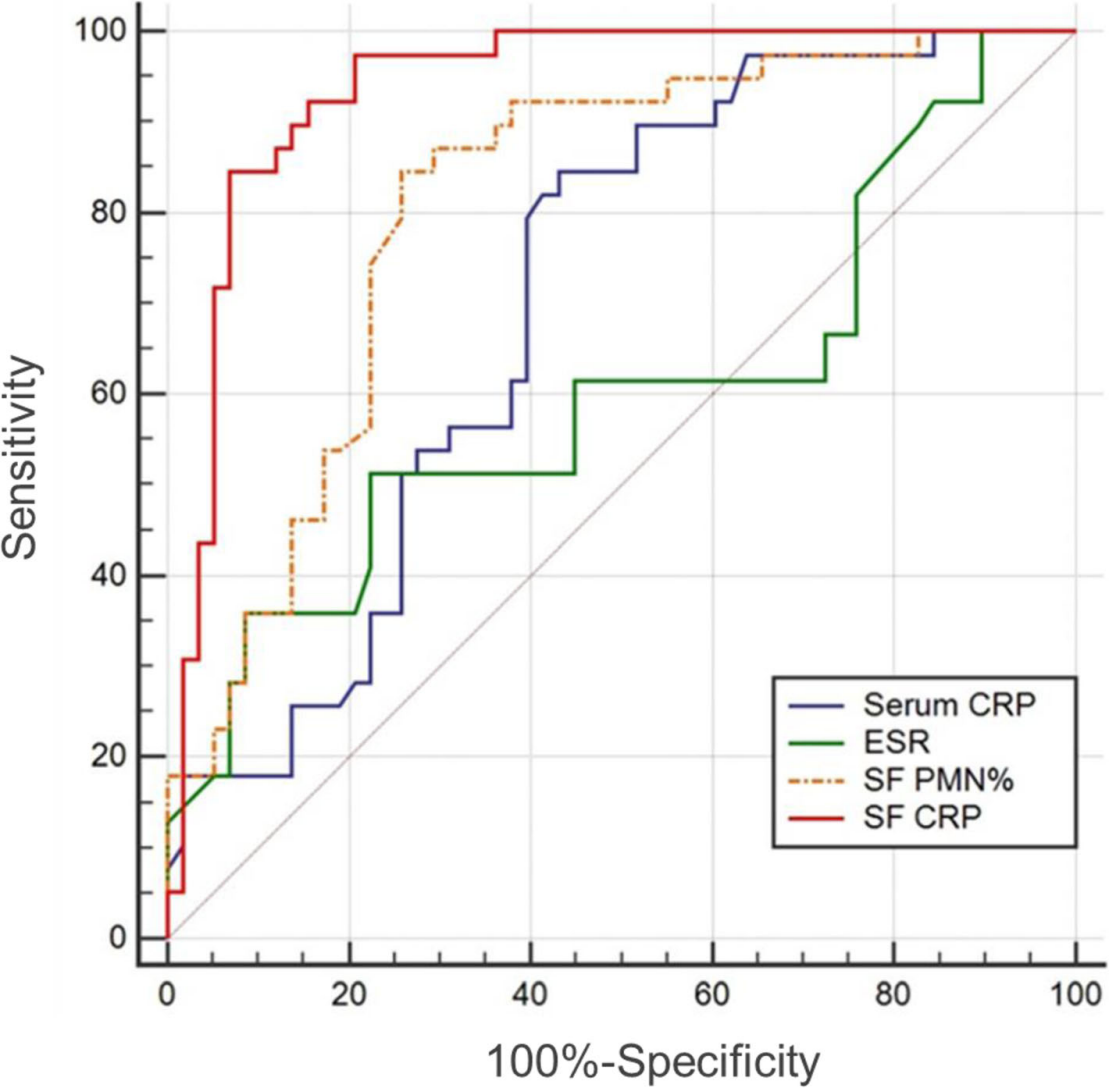
Receiver Operating Characteristic curves (ROCs). ROCs with the corresponding Area under the curve (AUC) of various inflammatory markers of patients with PJI or aseptic failure after TJA. CRP, C-reactive protein; ESR, erythrocyte sedimentation rate; SF, synovial fluid; SE, serum

To visualize the sensitivity and specificity of the measured biomarkers to predict the cause of revision (aseptic or infection), a conventional ROC curve and the area under the curve (AUC) were calculated (Fig. 3). The ROC curve analysis revealed the highest AUC for synovial fluid CRP, at 0.937 (95% confidence interval (95% CI), 0.869–0.976). Using Youden’s index, the optimal cut-off values were 7.26 mg/l, 10.2 mg/l, 69.79% and 34 mm/h, for synovial CRP, serum CRP, synovial PMN% and ESR, respectively, discriminating between PJI and aseptic failure.

As shown in Table 3, the synovial CRP level (7.26 mg/l) demonstrated a mean sensitivity of 84.62% (95% CI 69.5 to 94.1) and a mean specificity of 93.10% (95% CI 83.3 to 98.1%). The optimal serum CRP cut-off value was calculated at 10.02 mg/l, with sensitivity, specificity, and negative predictive value (NPV) of 84.62% (95% CI 69.5 to 94.1), 56.90% (95% CI 43.2 to 69.8), and 84.6% (95% CI 71.8 to 92.2), respectively. To a better evaluation of the combined application of serum and synovial CRP, we designed two combined models, model I (Serum CRP > 10.2 mg/l OR SF CRP > 7.26 mg/l) and model II (Serum CRP > 10.2 mg/l AND Synovial CRP > 7.26 mg/l), respectively. The combined model I (Serum CRP > 10.2 mg/l OR Synovial CRP > 7.26 mg/l) had a negative predictive value (NPV) of 96.67%, and a positive predictive value (PPV) of 56.72%. The combined model II (Serum CRP > 10.2 mg/l AND Synovial CRP > 7.26 mg/l) had an NPV of 84.06%, and a PPV of 1 (Table 3).

**Table 3:** Sensitivity, Specificity, PPV, NPV, and accuracy of inflammatory markers

Parameters	ESR (mm/h)	Serum CRP (mg/L)	Synovial PMN (%)	Synovial CRP (mg/L)	Serum CRP OR Synovial CRP (mg/L)	Serum CRP AND Synovial CRP (mg/L)
AUC (95%CI)	0.600 (0.495, 0.698)	0.703 (0.601, 0.791)	0.810 (0.718, 0.882)	0.937 (0.869, 0.976)	/	/
Cut-off level	34	10.2	69.79	7.26	Serum CRP > 10.2 OR Synovial CRP > 7.26	Serum CRP > 10.2 AND Synovial CRP > 7.26
Sensitivity (%) (95%CI)	51.28 (34.8, 67.6)	84.62 (69.5, 94.1)	84.62 (69.5, 94.1)	84.62 (69.5, 94.1)	97.44 (92.5, 100)	71.79 (57.7, 85.9)
Specificity (%) (95%CI)	77.59 (64.7, 87.5)	56.90 (43.2, 69.8)	74.14 (61.0, 84.7)	93.10 (83.3, 98.1)	50.00 (37.1, 62.9)	100 (/)
PPV (%)	60.6 (46.6, 73.1)	56.9 (48.8, 64.6)	68.7 (58.2,77.6)	89.2 (76.0, 95.5)	56.72 (44.9, 68.6)	100 (/)
NPV (%)	70.3 (62.5, 77.1)	84.6 (71.8, 92.2)	87.8 (77.2, 93.8)	90.0 (81.1, 95.0)	96.67 (90.2, 100)	84.06 (75.4, 92.7)
Accuracy (%)	67.01	68.04	78.35	89.69	69.07	88.66

*CRP* C-reactive protein, *ESR* erythrocyte sedimentation rate, *PMN%* Percentage of Polymorphonuclear Cells, *CI* confidence interval, *PPV* positive predictive value, *NPV* negative predictive value

## Discussion

In this prospective study, we analyzed and compared serum and synovial inflammatory factors in patients with chronic infection and aseptic after knee and hip arthroplasties. Our data indicate that serum and synovial CRP was significantly higher in the chronic infected group than in the aseptic group (19 mg/l vs 9.25 mg/l, and 9.93 mg/l vs 3.58 mg/l, *p* < 0.001, Table 2). We found a strong correlation between serum and synovial fluid CRP level (r = 0.523, *P* = .0012). This suggests that the circulatory system CRP is detected by spreading to the synovial fluid through increased vascular and synovial permeability due to infection [14, 19]. We determined that the serum CRP threshold for diagnosing chronic PJI was 10 mg/l, which was significantly lower than the results (39.8 mg/l) of a recent multicentric study conducted by Parvizi [20]. We found that when the threshold of synovial CRP was 7.26 mg/l, the AUC area of chronic PJI was as high as 93.70% (95%CI 0.869 to 0.976). However, the thresholds for synovial CRP that we determined were different from previous studies [14, 21] (2.8 mg/l to 9.5 mg/l), which may be due to reduced inflammatory response in chronic PJI patients with low-toxic infections, as well as differences in measurement.

Meanwhile, we also show that when serum CRP > 10.2 mg/l or synovial CRP > 7.26 mg/l, the diagnosed NPV value reached 96.67%. This combination can significantly improve diagnostic sensitivity, but specificity will be sacrificed. In our cohort, there will be 29 aseptic patients who will be misdiagnosed with an infection. This is due to the use of parallel tests that result in more patients being diagnosed positive and fewer being diagnosed negative. Therefore, the number of true positives increases (sensitivity increases) and the number of true negatives decreases (specificity decreases), which improves the misdiagnosis rate and reduces the rate of missed diagnosis. Accordingly, when serum and synovial CRP were both high than 10.2 mg/l and 7.26 mg/l, respectively, the diagnostic PPV value reached 1, and the specificity of diagnosis was improved to 1. This combination can be prepared to exclude non-infected patients and improve the efficiency of clinical diagnosis. To our knowledge, this is the first study to use different predictive models of serum and synovial CRP in the identification of chronic PJI.

The sensitivity of serum CRP reached 84.62% was in line with that of a previously published study, included 4934 participants, by Parvizi J et al. [22]. However, the serum CRP showed false-positive cases and, hence, a reduced specificity in our study, resulting in a potential overtreatment with unnecessary surgical revisions and prolonged antimicrobial treatment if used alone. The reasonable explanation is that CRP, a factor secreted by a variety of cells, increases to different degrees when the body is stimulated by inflammation [3, 6]. Therefore, we investigated inflammatory markers of local synovial fluid in joints with higher specificity [4]. Based on previous studies, detection time and economic benefits, we focused on the value of synovial CRP in differentiating chronic PJI [14, 23–25]. Our study indicated the synovial CRP possess a 93.10% diagnostic specificity and 89.69% diagnostic accuracy in identifying chronic PJI (Table 3). However, the analysis found that synovial-CRP sensitivity in differentiating chronic PJI was only 84.62%, ranging from 69.5 to 94.1%. A possible reason for the low sensitivity is the formation of mature biofilms in patients with chronic infection, which protect the pathogen against the host immune system resulting in a weakened immune response and, hence, reduced release of CRP [26, 27]. Therefore, we also do not recommend the use of synovial CRP alone for the diagnosis of chronic PJI.

To prevent this, we recommend the combined application of serum CRP and synovial CRP to timely detect chronic PJI. The current data suggested that the combination model I of serum and synovial CRP (Serum CRP > 10.2 OR Synovial CRP > 7.26) be first used to achieve higher diagnostic sensitivity and to reduce the false negative rate of diagnosis. And then using the combined model II of serum and synovial CRP (Serum CRP > 10.2 AND Synovial CRP > 7.26) to improve diagnostic specificity and thus reduce the false positive rate. The effectiveness of this joint diagnostic approach was recently recognized in a systematic review by Abdelbary et al. [28]. After this screening, histopathology and other tests can be used in the remaining patients to minimize missed and misdiagnosis. There was no consensus on the optimal thresholds of serum CRP alone and its combination with synovial CRP for the detection of chronic PJI. The current study established thresholds for serum and synovial CRP (10.2 mg/l and 7.26 mg/l, respectively), and developed two predictive models for the diagnosis of chronic PJI that were highly valuable. In addition, based on the premise of effectiveness, serum and synovial CRP have low cost-effectiveness, (USD $12 per test) and the detection ability of CRP is available in many hospitals. Therefore, this combination method has the potential to be widely used.

There were some limitations in the present study. First of all, there is no clear consensus on the exact time of postoperative chronic PJI. Although the time of biofilm maturation and previous published studies were used as references in this study, cases of acute infection in included chronic PJI patients could not be completely excluded [4, 29, 30]. Secondly, this study involved a single center, and the sample size was relatively small, with only 39 cases in the chronic PJI group and 58 cases in the aseptic group. However, this preliminary trial shows valuable results and warrants a larger multicentric study to verify the efficacy of serum and synovial CRP in the diagnosis of chronic PJI. Thirdly, in order to avoid the impact of other diseases on CRP levels, we excluded patients with inflammatory diseases, which would limit the application of the conclusions of this study. Finally, patients with recent antibiotic use were excluded from this study for the elimination of confounding factors. However, this may reduce the number of patients enrolled, limiting the generalizability of the results of this study.

## Conclusions

This is the first study concentrating on the diagnoses of chronic infections against aseptic failure of knee and hip arthroplasty. Synovial CRP is a valuable test that can well exclude chronic PJI through a combination of serum and synovial CRP (Serum CRP ≤ 10.2 mg/l AND Synovial CRP ≤ 7.26 mg/l). Therefore, this study is a valuable addition to the current diagnostic criteria developed by the International Consensus Conference on Musculoskeletal Infection (ICM) [31]. And we recommend that the synovial CRP test be considered for all patients suspected of PJI.

## Supplementary Information

**Additional file 1.**

## Data Availability

The datasets used or analyzed in the current study are available from the corresponding author up reasonable request.

## Abbreviations

PJI: Periprosthetic joint infection
CRP: C-reactive protein
ESR: Erythrocyte sedimentation rate
PMN%: Percentage of Polymorphonuclear Cells
SF: Synovial fluid
CI: Confidence interval
PPV: Positive predictive value
NPV: Negative predictive value
ROC: Receiver operating characteristic
AUC: Area under the curve
MSIS: Musculoskeletal Infection Society
WBC: White blood-cell
MRSA: Methicillin-resistant *S. aureus*
ICM: International Consensus Conference
