# Supplementary material for: Combined serum and synovial C-reactive protein tests: a valuable adjunct to the diagnosis of chronic prosthetic joint infection
Source: BMC Musculoskelet Disord. 2021 Aug 9;22:670. doi: 10.1186/s12891-021-04545-6 (PMC8353858; doi:10.1186/s12891-021-04545-6)
Supplement: Supplementary file 1 — Additional file 1. [file 12891_2021_4545_MOESM1_ESM.docx]

Supplementary Table 1. The Musculoskeletal Society 2013 definition of PJI

| MSIS definition of PJI* | |
| --- | --- |
| 1 | There is a sinus tract communicating with the prosthesis; or |
| 2 | Two positive periprosthetic cultures with phenotypically identical organisms; or |
| 3 | When 3 of the following 5 criteria exist:  a. Elevated serum C-reactive protein (CRP) AND erythrocyte sedimentation rate (ESR)  b. Elevated synovial fluid white blood cell (WBC) count OR ++change on leukocyte esterase test strip  c. Elevated synovial fluid polymorphonuclear neutrophil percentage (PMN%)  d. Positive histological analysis of periprosthetic tissue  e. A single positive culture |

*One of the three criteria (1, 2, or 3) must be met for diagnosis of periprosthetic joint infection.

Supplementary Table 2. Analysis of inflammatory markers in patients with infected revision arthroplasty

| Inflammatory marker | Infection | | |
| --- | --- | --- | --- |
|  | Hip (n=28) | Knee (n=11) | P value |
| ESR（mm/h） |  |  |  |
| median | 36.00 | 35.00 | 0.463^#^ |
| P25, P75 | (16.13, 50.00) | (12.00, 47.00) |  |
| Serum CRP (mg/L) |  |  |  |
| median | 18.70 | 19.00 | 0.920^#^ |
| P25, P75 | (13.20, 45.10) | (12.00, 32.20) |  |
| PMN% (%) |  |  |  |
| median | 78.75 | 73.44 | 0.729^#^ |
| P25, P75 | (71.42, 89.65) | (71.70, 91.57) |  |
| Synovial CRP (mg/L) |  |  |  |
| median | 9.89 | 9.93 | 0.836^#^ |
| P25, P75 | (8.34, 11.86) | (7.70, 14.45) |  |

“#” Mann-Whitney-U test; CRP, C-reactive protein; ESR, erythrocyte sedimentation rate; PMN%: Percentage of Polymorphonuclear Cell
